# Risk Factors for Steatorrhea in Chronic Pancreatitis: A Cohort of 2,153 Patients

**DOI:** 10.1038/srep21381

**Published:** 2016-02-15

**Authors:** Bai-Rong Li, Jun Pan, Ting-Ting Du, Zhuan Liao, Bo Ye, Wen-Bin Zou, Hui Chen, Jun-Tao Ji, Zhao-Hong Zheng, Dan Wang, Jin-Huan Lin, Shou-Bin Ning, Liang-Hao Hu, Zhao-Shen Li

**Affiliations:** 1 Department of Gastroenterology & Digestive Endoscopy Center, Changhai Hospital, The Second Military Medical University, Shanghai, China; 2 Department of Gastroenterology, Air Force General Hospital, Beijing, China

## Abstract

This study aimed to investigate the occurrence of and determine the risk factors for steatorrhea in chronic pancreatitis (CP). It was based on analysis of both retrospectively and prospectively acquired database for CP patients admitted to our center from January 2000 to December 2013. Demographic data, course of disease, medical history, and follow-up evaluations of patients were documented in detail. Cumulative rate of steatorrhea was calculated by using the Kaplan–Meier method. For risk factor analysis, multivariate analysis by Cox proportional hazards regression model was performed. A total of 2,153 CP patients were included with a mean follow-up duration of 9.3 years. Approximately 14% (291/2,153) of CP patients presented with steatorrhea at diagnosis of CP. Cumulative rates of steatorrhea at 1, 5, 10, and 20 years after diagnosis of CP were 4.27% (95% CI: 3.42%–5.34%), 12.53% (95% CI: 10.74%–14.59%), 20.44% (95% CI: 17.37%–23.98%) and 30.82% (95% CI: 20.20%–45.21%), respectively. Male gender (HR = 1.771, p = 0.004), diabetes (HR = 1.923, p < 0.001), alcohol abuse (HR = 1.503, p = 0.025) and pancreaticoduodenectomy (HR = 2.901, p < 0.001) were independent risk factors for steatorrhea while CP in adolescents (HR = 0.433, p = 0.009) was a protective factor. In conclusion, male gender, adult, diabetes, alcohol abuse and pancreaticoduodenectomy lead to increased risk of steatorrhea in CP patients.

Chronic pancreatitis (CP) is a progressive condition characterized by pancreatic acinar atrophy and fibrosis, which leads to irreversible damage of pancreatic endocrine and exocrine function. CP patients with pancreatic exocrine insufficiency (PEI) usually present with nutrition malabsorption which leads to vitamin and micronutrient deficiency and weight loss^1^,^2^, and have increased risks of developing premature atherosclerosis, cardiovascular events, osteoporosis, fracture, immune deficiency, and infection^3^,^4^,^5^.

It has been reported that 42%–99% of CP patients may develop PEI^6^,^7^,^8^,^9^,^10^. Although several direct and indirect function tests are available for the assessment of pancreatic function, diagnosis of mild/moderate PEI is difficult as these pancreatic function tests are either invasive or have limited diagnostic accuracy^11^,^12^. Steatorrhea, an overt presentation of severe PEI, is commonly observed in CP patients and occurs at the late stage of disease course when less than 10% of the pancreatic exocrine function is preserved^13^,^14^. Identifying the risk factors for steatorrhea might be helpful for indicating mild/moderate PEI.

Factors concerning disease duration and etiology might be associated with increased/decreased risk of developing steatorrhea. For example, steatorrhea is more common during the second decade after the onset of CP^7^. In alcoholic chronic pancreatitis (ACP), the interval between the first attack of a CP symptom and steatorrhea is around 13 years, which is substantially shorter than that in hereditary pancreatitis (HP) (≥26 years)^6^,^15^. The effect of pancreatic ductal morphology and calcification on the development of steatorrhea is currently a subject of debate^8^,^16^,^17^,^18^. Other patient-related factors, such as the initial symptom of CP, type of abdominal pain, severe acute pancreatitis attack, and treatment strategy, might also be related to steatorrhea development.

This study was based on a retrospective-prospective cohort of 2,153 CP patients with a long duration of follow-up after the onset of CP. We aimed to determine the prevalence of steatorrhea in CP patients and identify the risk factors, which might help to improve the outcome of CP patients with PEI.

## Results

### General characteristics of patients

**Figure 1:**
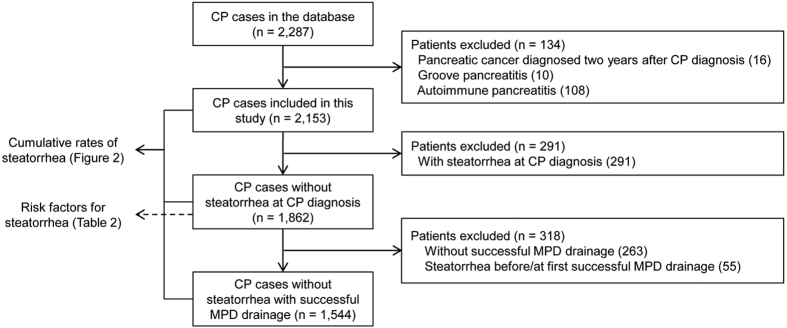
Different analyses employed for different patients.

From January 2000 to December 2013, a total of 2,287 CP patients were enrolled in Changhai CP Database. A total of 134 patients, which consisted of 16 patients diagnosed with pancreatic cancer within two years after the diagnosis of CP, 108 AIP patients, and 10 GP patients, were excluded from this study (Fig. 1). The general characteristics of the remaining 2,153 patients were presented in Table 1. We lost contact with 260 patients (12.1%) during follow-up, and the mean duration of follow-up was 9.3 years (SD 7.2 years). Idiopathic chronic pancreatitis (ICP) was most common (76.27%) in this study, whereas the proportion of ACP was 18.81% only. Among the 2,153 CP patients, a total of 493 (22.90%) developed steatorrhea during follow-up.

As Changhai Hospital is a tertiary medical center, almost 30% of the patients admitted to our center had received interventional procedures in primary medical centers. Minimally invasive interventions were used as principle methods prior to surgery in our center, and the overall treatment strategy was classified as endotherapy (including ESWL) alone (1524, 70.78%), surgery alone (236, 10.96%), both endotherapy and surgery (162, 7.52%), and no interventions due to lack of clinical symptoms (231, 10.73%). A total of 70 patients died during the follow-up period, and the causes of death were pancreatic cancer (19, 27.14%), complications of CP (17, 24.29%), non-pancreatic diseases (28, 40%), and accidental death (6, 8.75%).

### Clinical characteristics of steatorrhea and non-steatorrhea patients

Steatorrhea patients differed from non-steatorrhea patients in the following aspects: more male patients, fewer adolescent patients, higher prevalence of diabetes mellitus (DM), more ACP, more patients with initial manifestations of DM or steatorrhea, and different types of abdominal pain. No significant differences between these two groups were detected in terms of age at the onset or diagnosis of CP, common bile duct (CBD) stricture, pancreatic pseudocyst, stone, development of cancer, interventional treatment strategy, and death (Table 1).

### Cumulative rate of steatorrhea

#### After the onset of CP

**Figure 2:**
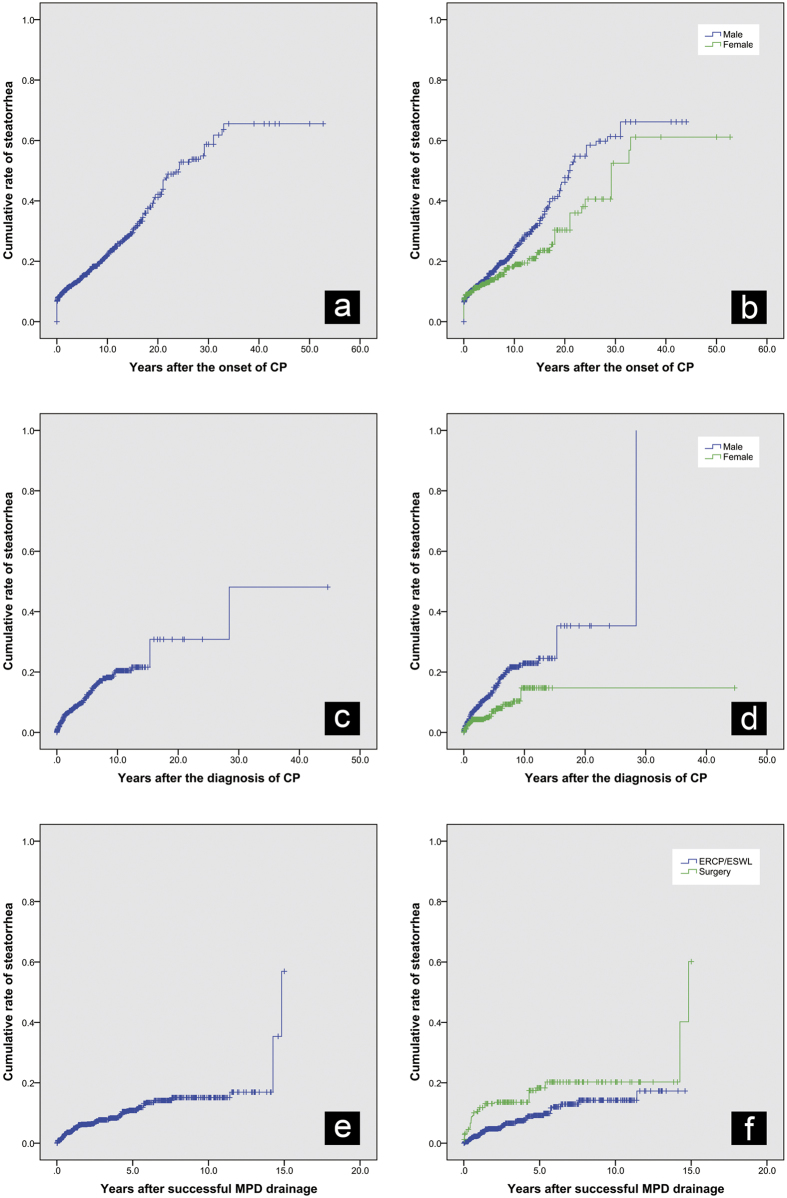
Cumulative rates of steatorrhea. (**a**) Overall cumulative rate of steatorrhea after the onset of chronic pancreatitis (CP); (**b**) Cumulative rates of steatorrhea stratified by gender (male vs. female) after the onset of CP; (**c**) Overall cumulative rate of steatorrhea after the diagnosis of CP; (**d**) Cumulative rates of steatorrhea stratified by gender (male vs. female) after the diagnosis of CP; (**e**) Overall cumulative rate of steatorrhea after successful main pancreatic duct (MPD) drainage; (**f**) Cumulative rates of steatorrhea stratified by method for MPD drainage (ERCP/ESWL vs. surgery).

Steatorrhea developed in 22.90% (493/2153) of the 2,153 eligible patients after the onset of CP; the rates were 24.83% in male patients (369/1486) and 19.19% in female patients (128/667). Steatorrhea developed in 200, 298, 381 and 466 patients at 1, 5, 10 and 20 years after the onset of CP, with the cumulative rates being 9.34% (95% CI: 8.18%–10.66%), 14.76% (95% CI: 13.27%–16.40%), 21.87% (95% CI: 19.89%–24.03%) and 41.14% (95% CI: 36.94%–45.62%), respectively. Moreover, a significant difference in the rate of steatorrhea was observed between male and female patients (p = 0.003, Fig. 2a,b).

#### After the diagnosis of CP

Fourteen percent (291/2153) of patients manifested with steatorrhea at the diagnosis of CP. After excluding these patients, the remaining 1,862 patients were included to calculate the cumulative rate of steatorrhea after the diagnosis of CP. Steatorrhea developed in 74, 165, 198 and 201 patients 1, 5, 10, and 20 years after the diagnosis of CP, with the cumulative rates being 4.27% (95% CI: 3.42%–5.34%), 12.53% (95% CI: 10.74%–14.59%), 20.44% (95% CI: 17.37%–23.98%) and 30.82% (95% CI: 20.20%–45.21%), respectively. Male patients had a significantly higher rate of steatorrhea compared with female patients (p < 0.001, Fig. 2c,d).

#### After the first successful MPD drainage

For 1,544 patients who showed no signs of steatorrhea when successful drainage of MPD was performed by interventions, the cumulative rate of steatorrhea after the first successful intervention that achieved MPD drainage was described. Steatorrhea developed in 54, 109 and 123 patients at 1, 5, and 10 years after intervention treatment, with the cumulative rates being 3.91% (95% CI: 3.00%–5.07%), 10.86% (95% CI: 8.93%–13.17%) and 15.09% (95% CI: 12.34%–18.39%), respectively (Fig. 2e). Among the 1,544 patients, 82.19% (1269/1544) achieved successful drainage of MPD via endotherapy and the remaining 17.81% (275/1544) via surgery. Log-rank test showed that endotherapy group had lower cumulative rate of steatorrhea than the surgery group (mean interval of steatorrhea: 12.93 years vs. 12.17 years, p < 0.001, Fig. 2f).

### Predictors for steatorrhea

All 20 variables in Table 2 were considered potential predictors of newly occurring steatorrhea, and were analyzed in the univariate analysis. Eight variables showed a *p* value less than 0.10. These eight variables were selected as candidates for multivariate analysis by the Cox proportional hazards regression model. The results showed that five factors were independent predictors of newly onset steatorrhea. The risk factors were male gender (hazard ratio (HR) = 1.771, p = 0.004), DM (HR = 1.923, p < 0.001), alcohol abuse (HR = 1.503, p = 0.025), and pancreaticoduodenectomy (HR = 2.901, p < 0.001) and the protective factor was CP in adolescents (HR = 0.433, p = 0.009).

## Discussion

PEI, which has a negative effect on nutrition absorption, is rarely confirmed at the early stage for CP patients. Severe PEI, namely, pancreatic exocrine failure, which manifests as clinical steatorrhea, is commonly diagnosed in CP patients. A substantial proportion of CP patients had PEI without overt steatorrhea, and detection of risk factors for PEI would be clinically important. But direct function tests available for PEI are invasive and with risk of complications, which makes them difficult to be used in clinical practice as well as in clinical research. For the current study, we focused on severe PEI, and presence of steatorrhea was set as the sign of severe PEI. Steatorrhea is related to increased risks of cardiovascular events, osteoporosis, fracture *et al.* and is a direct and decisive evidence of PEI for CP in clinical practice. We detected the prevalence of steatorrhea in CP and its predictors with a relatively large sample size (Table 3), which may help improve the outcome of CP patients with PEI.

For 2,153 CP patients, the overall incidence rate of steatorrhea was 22.9% over a median follow-up period of 7.8 years after the onset of CP. The cumulative rate of steatorrhea after the diagnosis of CP was 12.5% at 5 years, whereas Sandhu *et al.* reported a risk rate of 28% over a median follow-up period of 3.7 years (including 159 patients)^16^. We reported a lower rate of steatorrhea with longer disease course, which might be due to fewer ACP patients in our study. Pancreatic exocrine function is dependent on adequate acinar mass and function, and alcohol is toxic to the pancreas and is likely to severely damage the pancreatic parenchyma.

We identified several predictors for steatorrhea. Possible explanations for decreased risk of steatorrhea for CP in adolescents were: (1) compared to adult CP patients, adolescent CP patients have better preservation of pancreatic function and also better repair capacity after injury; (2) most of adolescent CP cases were followed up in less than 20 years, and we expect similar cumulative risk of steatorrhea with a long-term follow-up. A previous study also showed that exocrine and endocrine insufficiency developed more slowly in early-onset CP than in late-onset CP^6^. Therefore, adolescent CP patients had a reduced risk of steatorrhea compared to adult CP patients during the equivalent period of CP course.

Pancreaticoduodenectomy was identified as an independent risk factor for steatorrhea, while drainage of pancreatic duct (by ESWL/ERCP or LPJ) or partial resection of pancreas (head resection or tail resection) seemed not to increase the risk of steatorrhea. This indicates that steatorrhea as a sign of pancreatic exocrine function failure is due to diffuse and severe loss of pancreatic parenchyma. CP being a progressive disease with loss of pancreatic acinar tissue, minimally invasive method rather than resectional procedures may be a better choice given the advantage of preservation of pancreatic parenchyma^19^.

In the current study, ACP was considered when alcohol intake exceeded 80 g/d for male and 60 g/d for female for at least 2 years for CP patients in the absence of other causes, respectively. ACP patients showed a higher risk of steatorrhea, but the risk might be underestimated. There might be a proportion of patients considered as ICP who might probably have ACP. The Oriental population might be more sensitive to alcohol-related injuries than the Caucasian population because they tend to have a less-active aldehyde dehydrogenase, which is a key detoxifying enzyme for alcohol^20^,^21^.

Patients with DM showed higher risk for steatorrhea. Considerable evidence suggests important functional interactions between the exocrine and endocrine pancreas. The interactions occur at several regulatory levels, and the true dimension is still unknown^22^.

Pancreatic stone was analyzed as one of the potential predictors of steatorrhea, which was indicated in several studies^7^,^23^,^24^. However, we were unable to find a relationship between pancreatic stone and steatorrhea risk in the context of ductal drainage, which is in agreement with the findings of Lankisch *et al.*^18^. Stone in CP is commonly considered to be associated with an abnormal constitution of proteins and supersaturation of calcium carbonate in pancreatic juice. However, the detailed mechanism is still unknown, and the interaction between stone and PEI is poorly understood.

The Changhai CP Database established in 2005 has been collecting clinical data of CP patients since January 2000, and data have been collected retrospectively before January 2005 and prospectively ever since. Statistical analysis showed that there was no significant difference between the clinical characteristics of patients who were first admitted before January 2005 and those admitted after that date. Therefore, the recall bias minimally influenced the results of the study.

Our study has some limitations. First, clinical steatorrhea was used as a sign of severe PEI; dietary habits and celiac disease-related steatorrhea were not considered. Second, the retrospectively acquired data collected between 2000 and 2004 might introduce recall bias. Prospective data collection since January 2005 minimized the chance of incomplete or inaccurate data collection. Third, risk factor analysis did not include all potential factors related to the development of steatorrhea. Fourth, 603 CP patients were followed up for less than 2 years after the diagnosis of CP; among these patients, several pancreatic cancer patients may have been misdiagnosed as CP^25^. However, these limitations minimally influence the results considering the relatively large sample size of the study.

In conclusion, CP patients showed increased risk of steatorrhea for those of male gender, adults, DM, alcohol abuse and pancreaticoduodenectomy. The evaluation of risk factors in CP patients before the occurrence of steatorrhea might help determine the replacement therapy of pancreatic enzyme earlier, which ensures that severe complications related to PEI can be avoided. Prospectively conducted studies are expected to confirm the benefit of early treatment of PEI on CP patients.

## Methods

This study was based on analysis of both retrospectively and prospectively acquired database.

### Patients and database

Since the 1990s, an electronic medical record system (GOODWILL Inc., Beijing, China) has been used in Changhai Hospital (Shanghai, China), which has facilitated several studies on CP^26^,^27^,^28^,^29^. In order to track changes consistently throughout the course of CP and to facilitate the evaluation and study of the disease, a dedicated database, Changhai CP Database (version number 2.1, YINMA Information Technology Inc., Shanghai, China), was established in 2005 to collect clinical data of CP patients ever since. Data from January 2000 to December 2004 were retrospectively collected according to the electronic medical record system, and additional data were collected through telephone, letter, and e-mail inquiries. Data were prospectively collected since January 2005. The following information was documented in detail: demographic data (age, sex, birthplace, *et al.*), course of CP, medical history, history of other diseases, smoking history/status, alcohol history/status, family history of pancreatic diseases and DM, laboratory and imaging findings, and treatment strategy.

The database system was also set a reminder for investigators to call patients for clinical checkups. Aside from visits due to complaints of discomfort related to CP, all patients were periodically (at least once a year) recalled for clinical checkup and investigations. Ultrasound, MRI, or CT was selected as an evaluation modality during each follow-up visit. An evaluation of each revisit, or an evaluation via telephone inquiries for patients who did not have follow-up visits to Changhai Hospital, was added to the CP database.

In December 2013, we contacted all the patients in our database for a final evaluation, except those who were lost to follow-up or died. The duration of follow-up is defined as the duration from the onset of CP to the date of the last personal contact, death, or December 2013, whichever came first.

The study was approved by the Ethics Committee of Changhai Hospital, The Second Military Medical University, Shanghai, China according to the Helsinki Declaration. Written informed consent was obtained from all participating patients. All of the diagnostic and therapeutic modalities were carried out in accordance to the approved guidelines.

### Definitions

CP was diagnosed according to the Asia-Pacific consensus of CP^30^. ACP was diagnosed when alcohol intake exceeded 80 g/d for male and 60 g/d for female for at least two years in the absence of other causes, respectively^27^,^31^. HP was diagnosed when the CP patient had no less than two first-degree relatives with CP or recurrent acute pancreatitis (AP), or no less than three second-degree relatives with CP or recurrent AP^32^. We defined abnormal anatomy of pancreatic duct system (including pancreas divisum and anomalous pancreaticobiliary junction) as an etiology of CP in our study, although it still remains a controversy^33^. A patient was defined as post-traumatic CP due to a definite history of abdominal trauma with imaging evidence of pancreatic injury and subsequent ductal dilation. Hyperlipidemia was considered as an etiology when blood triglyceride was higher than 1,000 mg/dL at the diagnosis of CP^34^. CP patients were considered idiopathic when none of the above etiologies were found.

Patients who were diagnosed with pancreatic cancer less than two years after the diagnosis of CP were excluded from this study^26^,^35^. Autoimmune pancreatitis (AIP) and groove pancreatitis (GP) were also excluded from this study: AIP is different from typical CP in terms of pathophysiology, clinical manifestations, and prognosis, while GP could hardly be differentiated from pancreatic head carcinoma until pancreaticoduodenectomy and confirmed histological findings^36^.

Diagnosis of steatorrhea was established when either of the following conditions was met: (1) chronic diarrhea with foul-smelling, oily bowel movements^37^; (2) a positive result in quantification of fecal fat determination (fecal fat quantification was performed over a period of three days; steatorrhea was defined as a fecal fat excretion of more than 14 g/day).

### Treatment strategy

As a tertiary medical center, Changhai Hospital admitted patients with previous pancreas-related surgical, endoscopic, or other invasive procedures from primary medical centers. In our center, minimally invasive interventions were taken as principle methods prior to surgery: extracorporeal shock wave lithotripsy (ESWL)/endoscopic retrograde cholangiopancreatography (ERCP) for stone removal and main pancreatic duct (MPD) drainage, insertion of stents to treat dominant MPD stricture and biliary duct stricture, and endoscopic drainage for uncomplicated pseudocyst with endoscopic reach^28^,^35^,^38^,^39^,^40^,^41^,^42^,^43^,^44^,^45^. For CP patients who did not experience pain, interventions were performed only when CP was complicated by CBD stricture, pancreatic portal hypertension, *et al.*; DM or steatorrhea was not an indication for invasive treatment of CP^13^.

### Data management and statistical analysis (Fig. 1)

Continuous and categorical variables were presented as mean ± SD and counts (percentages), respectively. Student’s t-test or Mann–Whitney U test and χ-square test or Fisher’s exact test were used as indicated. Cumulative rates of steatorrhea were calculated by using the Kaplan–Meier method after the onset of CP (all CP patients were included), after the diagnosis of CP (patients with steatorrhea at the diagnosis of CP were excluded), and after the first successful drainage of MPD (only patients who without steatorrhea at the time of the first successful drainage of MPD were included). Log-rank test was used to further analyze the difference of cumulative rates of steatorrhea between two groups (e.g., male vs. female, endotherapy vs. surgical treatment). For risk factor analysis, multivariate analysis by Cox proportional hazards regression model was performed to identify the independent predictors based on the results of univariable analyses (factors with a significance level of p < 0.10 were included in the multivariate analysis). Hazard ratios (HRs) and 95% confidence intervals (CIs) were calculated. Statistical analyses were conducted at a significance level of 0.05 for all analyses. Data were analyzed by using SPSS 18.0 (SPSS, Chicago, IL, USA).

## Additional Information

**How to cite this article**: Li, B.-R. *et al.* Risk Factors for Steatorrhea in Chronic Pancreatitis: A Cohort of 2,153 Patients. *Sci. Rep.* **6**, 21381; doi: 10.1038/srep21381 (2016).

## Figures and Tables

**Table 1:** General characteristics of 2,153 CP patients.

	Overall	Steatorrhea group	No steatorrhea group	*p*
Average Age. CP onset (SD) (years)	38.23(16.60)	37.05(13.65)	38.58(17.37)	0.042
Average Age. CP diagnosis (SD) (years)	43.08(15.54)	43.54(11.93)	42.94(16.46)	0.377
Median. follow-up duration (range) (months)	93.04(0–638.40)	109.05(1.20–638.40)	88.27(0–632.45)	<0.001
Male gender (%)	1,486(69.02%)	365(74.04%)	1,121(67.53%)	0.006
Steatorrhea (%)	493(22.90%)	493(100.00%)	0(00.00%)	
Adolescent (%)	291(13.52%)	46(09.33%)	245(14.76%)	0.002
Etiology (%)				<0.001
ACP*	405(18.81%)	121(24.54%)	284(17.11%)	
ICP	1,642(76.27%)	341(69.17%)	1,301(78.37%)	
Abnormal anatomy of pancreatic duct	64(02.97%)	15(03.04%)	49(02.95%)	
HCP	30(01.39%)	14(02.84%)	16(00.96%)	
Post-traumatic CP	10(00.46%)	2(00.41%)	8(00.48%)	
Hyperlipidemic CP	2(00.09%)	0(00.00%)	2(00.12%)	
Initial manifestation (%)				<0.001
Abdominal pain	1,796(83.42%)	332(67.34%)	1,464(88.19%)	
DM/steatorrhea	218(10.13%)	125(25.35%)	93(05.60%)	
Others	139(06.46%)	36(07.30%)	103(06.20%)	
Type of abdominal pain# (%)				<0.001
RAP	681(31.63%)	125(25.35%)	556(33.49%)	
RP	639(29.68%)	157(31.85%)	482(29.04%)	
RAP/P	570(26.47%)	113(22.92%)	457(27.53%)	
CPP	106(04.92%)	28(05.68%)	78(04.70%)	
No pain attack	157(07.29%)	70(14.20%)	87(05.24%)	
Pancreatic stone+ (%)	1,627(75.57%)	394(79.92%)	1,233(74.28%)	0.010
DM (%)	616(28.61%)	211(42.80%)	405(24.40%)	<0.001
CBD stenosis (%)	340(15.79%)	78(15.82%)	262(15.78%)	0.984
PPC formation (%)	350(16.26%)	73(14.81%)	277(16.69%)	0.321
Alcohol consumption (%)				0.001
No	1,426(66.23%)	294(59.63%)	1,132(68.19%)	
>0 g/d, <20 g/d	70(03.25%)	19(03.85%)	51(03.07%)	
≥20 g/d, <80 g/d	237(11.01%)	56(11.36%)	181(10.90%)	
≥80 g/d	420(19.51%)	124(25.15%)	296(17.83%)	
Smoking history (%)	723(33.58%)	177(35.90%)	546(32.89%)	0.214
Treatment strategy				0.085
No interventions	231(10.73%)	41(8.32%)	190(11.45%)	
Endotherapy	1,524(70.78%)	348(70.59%)	1,176(70.84%)	
Surgery	236(10.96%)	58(11.76%)	178(10.72%)	
Both endotherapy and surgery	162(7.52%)	46(9.33%)	116(6.99%)	

Abbreviations: CP, chronic pancreatitis; ACP, alcoholic chronic pancreatitis; ICP, idiopathic chronic pancreatitis; DM, diabetes mellitus; HCP, heredity chronic pancreatitis; RAP, recurrent acute pancreatitis; RAP/P, recurrent acute pancreatitis or adominal pain without significant increasing in serum amylase; CPP, chronic pancreatic pain; CBD, common bile duct; PPC, pancreatic pseudocyst.

^*^ACP is defined as 80 g/d alcohol consumption that lasted for no less than two years for men and 60 g/d for women. A total of 727 patients (33.8%) had a history of alcohol consumption.

^#^Referring to pain type in the course from onset to last personal contact or death.

^+^Pancreatic calcification was also regarded as stone(s) that located in branch pancreatic duct or ductulus^46^.

**Table 2:** Predictive factors for steatorrhea.

Predictive factor	n (%)	Univariate analysis		Multivariate analysis	
		HR (95% CI)	*P*	HR (95% CI)	*P*
Age at the onset of CP	38.46 ± 16.96*	0.999 (0.991–1.007)	0.739		
Adolescent	256 (13.75%)	0.309 (0.167–0.570)	<0.001	0.433 (0.231–0.811)	0.009
Age at the diagnosis of CP	42.83 ± 16.04*	1.000 (0.992–1.009)	0.962		
Age at the diagnosis of CP (<30, 30–40, 40–50, 50–60, ≥60)	–	0.934 (0.842–1.036)	0.198		
Gender (male)	1,286 (69.07%)	2.069 (1.447–2.959)	<0.001	1.771 (1.195–2.623)	0.004
Alcohol abuse#	341 (18.31%)	1.769 (1.301–2.404)	<0.001	1.503 (1.053–2.145)	0.025
Smoking history	623 (33.46%)	1.363 (1.025–1.813)	0.033		
Abnormal anatomy of pancreatic duct	54 (2.90%)	0.761 (0.313–1.848)	0.546		
Hereditary CP	18 (0.97%)	1.233 (0.306–4.968)	0.768		
Pancreatic disease in three-class relatives (excluding hereditary CP)	28 (1.50%)	0.186 (0.023–1.524)	0.117		
DM in three-class relatives	105 (5.64%)	0.571 (0.235–1.391)	0.217		
DM	273 (14.66%)	1.990 (1.431–2.767)	<0.001	1.923 (1.364–2.713)	<0.001
Pancreatic stone	1,246 (66.92%)	0.969 (0.727–1.291)	0.829		
Pancreatic stone status					
No stone	616 (33.08%)	Reference			
Peripheral ductal stones	139 (7.47%)	0.657 (0.347–1.246)	0.198		
MPD stone with/without concurrent peripheral ductal stones	1,107 (59.45%)	0.897 (0.664–1.212)	0.479		
Biliary stricture	133 (7.14%)	1.328 (0.818–2.155)	0.252		
Pancreatic pseudocysts	134 (7.20%)	0.760 (0.414–1.397)	0.377		
Abdominal pain	1,700 (91.30%)	0.584 (0.386–0.881)	0.010		
SAP	57 (3.06%)	0.276 (0.068–1.111)	0.070		
Successful drainage+	564 (30.29%)	1.123 (0.831–1.519)	0.450		
Treatment			0.004		
Conservative treatment	1,298 (69.71%)	Reference			
ERCP/ESWL	412 (22.13%)	0.841 (0.575–1.229)	0.371		
Pancreaticojejunostomy	86 (4.62%)	1.022 (0.326–3.211)	0.970		
Combined pancreaticojejunostomy and pancreatectomy	8 (0.43%)	∞	0.977		
Pancreaticoduodenectomy	21 (1.13%)	3.241 (2.116–4.965)	<0.001	2.901 (1.873–4.494)	<0.001
Distal pancreatectomy	28 (1.50%)	∞	0.941		
Other surgical procedures	9 (0.48%)	∞	0.974		

Abbreviations: CP, chronic pancreatitis; HR, hazard ratio; DM, diabetes mellitus; SAP, severe acute pancreatitis.

^*^Mean ± SD for continuous variables.

^#^Diagnosis criteria for alcoholic CP was used as a measure for alcohol consumption.

^+^Patients with successful main pancreatic duct (MPD) drainage are those whose CP was established after ERCP or pancreatic surgery or those who underwent successful MPD drainage during administration when CP diagnosis was established.

**Table 3:** Researches on risk factors of PEI in CP patients.

Authors (Year)	Design	Sample size	Period of follow-up	Method for evaluation of PEI	Number of factors included
Wakabayashi, A. *et al.* (1977)	Cross-sectional	19	NA	SPT	1
Braganza, J. M. *et al.* (1982)	Cross-sectional	45	NA	SPT	1
Ammann, R. W. *et al.* (1984)	Prospective	245	10.4 years [median]	NA	1
Lankisch, P. G. *et al.* (1986)	Cross-sectional	79	NA	SPT and fecal analysis	1
Hayakawa, T. *et al.* (1992)	Cross-sectional	108	NA	CST	1
Ammann, R.W. *et al.* (1996)	Prospective	73	12.0 years [median]	Fecal analysis	4
Sandhu, B.S. *et al.* (2007)	Retrospective	159	3.7 years [median]	Presence of steatorrhea	5
Dominguez-Muñoz JE. *et al.* (2012)	Cross-sectional	128	NA	Carbon 13-mixed triglyceride breath test	10
Li BR, *et al.* (2015)	Prospective	2,153	7.8 years [median]	Presence of steatorrhea and fecal fat analysis	18

Abbreviations: SPT, secretin-pancreozymin test; CST, cholecystokinin secretin test; NA, not available.

## References

[b1] Dominguez-Munoz J. E., Iglesias-Garcia J., Vilarino-Insua M. & Iglesias-Rey M. 13C-mixed triglyceride breath test to assess oral enzyme substitution therapy in patients with chronic pancreatitis. Clin Gastroenterol Hepatol. 5, 484–488 (2007). 17445754 10.1016/j.cgh.2007.01.004

[b2] Dominguez-Munoz J. E. & Iglesias-Garcia J. Oral pancreatic enzyme substitution therapy in chronic pancreatitis: is clinical response an appropriate marker for evaluation of therapeutic efficacy? JOP. 11, 158–162 (2010). 20208327

[b3] Pongprasobchai S. Maldigestion from pancreatic exocrine insufficiency. J Gastroenterol Hepatol. 28 Suppl 4, 99–102 (2013). 24251713 10.1111/jgh.12406

[b4] Montalto G. *et al.* Lipoproteins and chronic pancreatitis. Pancreas. 9, 137–138 (1994). 8108368 10.1097/00006676-199401000-00021

[b5] Tignor A. S. *et al.* High prevalence of low-trauma fracture in chronic pancreatitis. Am J Gastroenterol. 105, 2680–2686 (2010). 20736937 10.1038/ajg.2010.325

[b6] Layer P. *et al.* The different courses of early- and late-onset idiopathic and alcoholic chronic pancreatitis. Gastroenterology. 107, 1481–1487 (1994). 7926511 10.1016/0016-5085(94)90553-3

[b7] Ammann R. W., Akovbiantz A., Largiader F. & Schueler G. Course and outcome of chronic pancreatitis. Longitudinal study of a mixed medical-surgical series of 245 patients. Gastroenterology. 86, 820–828 (1984). 6706066

[b8] Braganza J. M., Hunt L. P. & Warwick F. Relationship between pancreatic exocrine function and ductal morphology in chronic pancreatitis. Gastroenterology. 82, 1341–1347 (1982). 7067955

[b9] Lankisch M. R., Imoto M., Layer P. & DiMagno E. P. The effect of small amounts of alcohol on the clinical course of chronic pancreatitis. Mayo Clin Proc. 76, 242–251 (2001). 11243270 10.4065/76.3.242

[b10] Ammann R. W., Buehler H., Muench R., Freiburghaus A. W. & Siegenthaler W. Differences in the (Nonalcoholic) and Natural History of Idiopathic Alcoholic Chronic Pancreatitis. A Comparative Long-Term Study of 287 Patients. Pancreas. 2, 368–377 (1987). 3628234 10.1097/00006676-198707000-00002

[b11] Nojgaard C., Olesen S. S., Frokjaer J. B. & Drewes A. M. Update of exocrine functional diagnostics in chronic pancreatitis. Clin Physiol Funct Imaging. 33, 167–172 (2013). 23522009 10.1111/cpf.12011

[b12] Keller J., Aghdassi A. A., Lerch M. M., Mayerle J. V. & Layer P. Tests of pancreatic exocrine function - clinical significance in pancreatic and non-pancreatic disorders. Best Pract Res Clin Gastroenterol. 23, 425–439 (2009). 19505669 10.1016/j.bpg.2009.02.013

[b13] Mergener K. & Baillie J. Chronic pancreatitis. Lancet. 350, 1379–1385 (1997). 9365465 10.1016/S0140-6736(97)07332-7

[b14] DiMagno E. P., Go V. L. & Summerskill W. H. Relations between pancreatic enzyme ouputs and malabsorption in severe pancreatic insufficiency. N Engl J Med. 288, 813–815 (1973). 4693931 10.1056/NEJM197304192881603

[b15] Ammann R. W. Diagnosis and management of chronic pancreatitis: current knowledge. Swiss medical weekly. 136, 166 (2006). 16633964 10.4414/smw.2006.11182

[b16] Sandhu B. S. *et al.* Recurrent flares of pancreatitis predict development of exocrine insufficiency in chronic pancreatitis. Clin Gastroenterol Hepatol. 5, 1085–1091; quiz 1007 (2007). 17588823 10.1016/j.cgh.2007.04.011

[b17] Hayakawa T. *et al.* Relationship between pancreatic exocrine function and histological changes in chronic pancreatitis. Am J Gastroenterol. 87, 1170–1174 (1992). 1519575

[b18] Lankisch P. G., Otto J., Erkelenz I. & Lembcke B. Pancreatic calcifications: no indicator of severe exocrine pancreatic insufficiency. Gastroenterology. 90, 617–621 (1986). 3943692 10.1016/0016-5085(86)91115-7

[b19] Dumonceau J. M. *et al.* Endoscopic treatment of chronic pancreatitis: European Society of Gastrointestinal Endoscopy (ESGE) Clinical Guideline. Endoscopy. 44, 784–800 (2012). 22752888 10.1055/s-0032-1309840

[b20] Perez-Miller S. *et al.* Alda-1 is an agonist and chemical chaperone for the common human aldehyde dehydrogenase 2 variant. Nat Struct Mol Biol. 17, 159–164 (2010). 20062057 10.1038/nsmb.1737 PMC2857674

[b21] Chan A. W. Racial differences in alcohol sensitivity. Alcohol Alcohol. 21, 93–104 (1986). 2937417

[b22] Nunes A. C. *et al.* Screening for pancreatic exocrine insufficiency in patients with diabetes mellitus. Am J Gastroenterol. 98, 2672–2675 (2003). 14687815 10.1111/j.1572-0241.2003.08730.x

[b23] Scuro L. A. *et al.* Pancreatic calcifications in patients with chronic pancreatitis. Int J Pancreatol. 6, 139–150 (1990). 2230361

[b24] Bhasin D. K. *et al.* Clinical profile of calcific and noncalcific chronic pancreatitis in north India. J Clin Gastroenterol. 45, 546–550 (2011). 20962669 10.1097/MCG.0b013e3181f8c6bf

[b25] Wang W. *et al.* Incidence of pancreatic cancer in chinese patients with chronic pancreatitis. Pancreatology. 11, 16–23 (2011). 21311209 10.1159/000322982

[b26] Wang W. *et al.* Incidence of pancreatic cancer in chinese patients with chronic pancreatitis. Pancreatology. 11, 16–23 (2011). 21311209 10.1159/000322982

[b27] Wang W. *et al.* Occurrence of and risk factors for diabetes mellitus in Chinese patients with chronic pancreatitis. Pancreas. 40, 206–212 (2011). 21404458 10.1097/mpa.0b013e31820032ae

[b28] Li Z. S. *et al.* A long-term follow-up study on endoscopic management of children and adolescents with chronic pancreatitis. Am J Gastroenterol. 105, 1884–1892 (2010). 20216535 10.1038/ajg.2010.85

[b29] Wang W. *et al.* Chronic pancreatitis in Chinese children: etiology, clinical presentation and imaging diagnosis. J Gastroenterol Hepatol. 24, 1862–1868 (2009). 19793170 10.1111/j.1440-1746.2009.05967.x

[b30] Tandon R. K., Sato N. & Garg P. K. Chronic pancreatitis: Asia–Pacific consensus report. J Gastroenterol Hepatol. 17, 508–518 (2002). 11982735 10.1046/j.1440-1746.2002.02762.x

[b31] Witt H. *et al.* A degradation-sensitive anionic trypsinogen (PRSS2) variant protects against chronic pancreatitis. Nat Genet. 38, 668–673 (2006). 16699518 10.1038/ng1797 PMC2746914

[b32] Howes N. *et al.* Clinical and genetic characteristics of hereditary pancreatitis in Europe. Clin Gastroenterol Hepatol. 2, 252–261 (2004). 15017610 10.1016/s1542-3565(04)00013-8

[b33] Lu W.-F. ERCP and CT diagnosis of pancreas divisum and its relation to etiology of chronic pancreatitis. World J Gastroenterol. 4, 150–152 (1998). 11819261 10.3748/wjg.v4.i2.150 PMC4688639

[b34] Yadav D. & Pitchumoni C. Issues in hyperlipidemic pancreatitis. J Clin Gastroenterol. 36, 54–62 (2003). 12488710 10.1097/00004836-200301000-00016

[b35] Li B. R., Hu L. H. & Li Z. S. Chronic pancreatitis and pancreatic cancer. Gastroenterology. 147, 541–542 (2014). 24979529 10.1053/j.gastro.2014.03.054

[b36] Malde D. J., Oliveira-Cunha M. & Smith A. M. Pancreatic carcinoma masquerading as groove pancreatitis: case report and review of literature. JOP. 12, 598–602 (2011). 22072250

[b37] Affronti J. Chronic pancreatitis and exocrine insufficiency. Prim Care. 38, 515–537; ix (2011). 21872095 10.1016/j.pop.2011.05.007

[b38] Sun X. T. *et al.* Clinical Features and Endoscopic Treatment of Chinese Patients With Hereditary Pancreatitis. Pancreas. 44, 59–63 (2015). 25058887 10.1097/MPA.0000000000000198

[b39] Li B. R. *et al.* Risk factors for complications of pancreatic extracorporeal shock wave lithotripsy. Endoscopy. 46, 1092–1100 (2014). 25251205 10.1055/s-0034-1377753

[b40] Hu L. H. *et al.* Extracorporeal shock wave lithotripsy as a rescue for a trapped stone basket in the pancreatic duct. Endoscopy. 46 Suppl 1 UCTN, E332–333 (2014). 25090466 10.1055/s-0034-1377221

[b41] He Y. X. *et al.* Endoscopic management of early-stage chronic pancreatitis based on M-ANNHEIM classification system: a prospective study. Pancreas. 43, 829–833 (2014). 24717828 10.1097/MPA.0000000000000140

[b42] Zou W. B., Hu L. H., Liao Z. & Li Z. S. Obscure hemosuccus pancreaticus due to dorsal pancreatic arteriorrhexis. Dig Liver Dis. 45, 346 (2013). 23137794 10.1016/j.dld.2012.09.020

[b43] Hu L.-H., Liao Z. & Li Z.-S. Rolling in the deep: a quaint sphere rolling in the deep pancreatic duct. Gastroenterology. 145, e7–e8 (2013). 24176871 10.1053/j.gastro.2013.07.007

[b44] Hu L. H., Liao Z. & Li Z. S. Spontaneous clearance of pancreatic stones. Clin Gastroenterol Hepatol. 11, e9–10 (2013). 22835576 10.1016/j.cgh.2012.07.005

[b45] Ceppa E. P. *et al.* Hereditary pancreatitis: endoscopic and surgical management. J Gastrointest Surg. 17, 847-856; discussion 856–847 (2013). 10.1007/s11605-013-2167-8 23435738

[b46] Ammann A. R. W. *et al.* Evolution and regression of pancreatic calcification in chronic pancreatitis. A prospective long-term study of 107 patients. Gastroenterology. 95, 1018–1028 (1988). 3410215 10.1016/0016-5085(88)90178-3

